# Smoking Avoidance, Physical Activity and Diet as Preventative Behaviours for Lung, Prostate and Colorectal Cancer - A Comparison of the Extended Parallel Process Model Groups

**DOI:** 10.3389/ijph.2025.1607278

**Published:** 2025-02-04

**Authors:** Katarzyna Domosławska-Żylińska, Dorota Włodarczyk

**Affiliations:** ^1^ Department of Education and Communication, National Institute of Public Health NIH - National Research Institute, Warsaw, Poland; ^2^ Department of Health Psychology, Medical University of Warsaw, Warsaw, Poland

**Keywords:** oncology, lung cancer, prostate cancer, colon cancer, diet, physical activity, EPPM

## Abstract

**Objectives:**

An analysis of men’s perceptions of the role of three health behaviours (smoking avoidance, physical activity, and diet) in relation to the subjective threat of lung, prostate, and colorectal cancers, with adoption of the Extended Parallel Process Model (EPPM).

**Methods:**

The study was conducted using a survey questionnaire by Computer Assisted Web Interviewing on a representative sample of 1,000 male Polish citizens aged 18–65.

**Results:**

Prostate cancer was considered the most likely and most severe type of cancer. A healthy diet was the intervention that was considered the most effective and the one most likely to be implemented for colorectal cancer. Respondents perceived smoking avoidance to be the most effective intervention, while considering this to be the least feasible strategy to implement for lung cancer. In all of the behaviours, the Indifferent group was the most numerous. Belonging to the EPPM groups was mainly associated with educational level, financial situation, and self-assessed health status.

**Conclusion:**

The need to implement interventions aimed at: increase the perceived risk of smoking in the context of lung cancer incidence, increase men’s self-efficacy in smoking avoidance and reduce the level of perceived losses from undertaking a healthy diet and smoking avoidance.

## Introduction

In 2019, almost 1.2 million people died from cancer in European Union (EU) countries, which accounts for more than a quarter (26%) of all deaths [1]. Poland is one of the EU countries that have cancer mortality rates more than 15% higher than the EU average [2–4]. In Poland in 2020, the most prevalent cancer among men was prostate cancer, which was responsible for 20.6% of all cases and 10.3% of all deaths, while lung cancer was responsible for 16.1% and 27.4%, respectively, and colorectal cancer was responsible for 12.2% and 13%, respectively [5]. The susceptibility to chronic diseases is influenced by modifiable risk factors (low physical activity, unhealthy diet, and smoking). For example, an unhealthy diet was linked to a 28% higher risk of colon cancer in men and the risk of developing lung cancer is about 26 times higher in men who smoke 15–24 cigarettes a day compared to those who have never smoked [6, 7]. The above results show how unhealthy lifestyle factors significantly affect the risk of cancer [8]. The risk of many types of cancer can be reduced through behaviour and lifestyle changes [9, 10]. However, not all health behaviours have the same effectiveness for preventing every cancer. When selecting these behaviours, we based both on global and national evidence [3]. To date, the effectiveness of non-smoking for lung cancer risk [11], physical activity for colorectal cancer risk [12], and diet for colorectal and prostate cancer risk has been confirmed to the highest degree [13, 14].

Two predictors included in many behaviour change theories are important for increasing the effectiveness of implementing behaviour change in cancer prevention: the perceived threat of cancer and different aspects of efficacy (like intervention efficacy and self-efficacy) [15]. Both a strong cancer threat and a highly rated efficacy may influence more frequent use of health behaviours and reduce cancer risk [16, 17]. One theory of health behaviours that focuses on the dimensions mentioned above is the Extended Parallel Process Model (EPPM). The perceived threat consists of an assessment of the extent to which the individual believes an adverse event is likely to occur (susceptibility) and how they perceive the severity of the event (severity) [18]. The perceived threat of cancer may motivate individuals to avoid risky health behaviours [19]. The second predictor in the EPPM is efficacy, consisting of the perceived efficacy of the behaviours themselves in preventing cancer (response efficacy) and the individual’s own efficacy in implementing health behaviours (self-efficacy). Research has shown that self-efficacy is associated with cancer prevention behaviours such as giving up smoking, seeking information about cancer, exercise, and healthy eating [15]. Based on these predictors, the EPPM classifies individuals into four groups (Supplementary Figure S1): Indifferent (low threat, low efficacy), Proactive (low threat, high efficacy), Avoidant (high threat, low efficacy), and Responsive (high threat, high efficacy). Examining differences between these groups can provide important information on strategic directions for effective cancer risk communication among men to increase the uptake of preventive behaviours.

The main aim of the study was to analyse men’s perceptions of the role of three health behaviours (smoking avoidance, physical activity, and diet) in relation to three cancers with different locations: lung, prostate, and colorectal cancers. These analyses included the parameters considered in the EPPM. The following specific objectives were formulated: 1) an analysis of the perceptions of smoking avoidance in the context of lung cancer, 2) an analysis of the perceptions of the role of diet and physical activity in relation to colorectal cancer, and 3) the perceptions of the role of diet in relation to prostate cancer.

## Methods

### Persons Surveyed

A representative sample of 1,000 male Polish citizens aged 18–65 took part in the study. The sample size was calculated using a sampling calculator with the following assumptions: a population size of 18,095,040 men according to the Central Statistical Office (2022), a confidence level of 98%, and a maximum error of 4%. According to this calculation the sample should contain at least 846 respondents. The selection of respondents reflected the age structure of the population in relation to the administrative division of the country and the size of the place of residence. The inclusion criteria were gender (male), age (adults 18–65 years from the general non-clinical population), and consent to participate in the study. There were no exclusion criteria.

### Study Design

The design phase of the study included an analysis of cancer mortality among Polish men, the selection of malignancies with the highest death rates among Polish men (lung cancer, colorectal cancer, and prostate cancer), and choosing health behaviours that are part of a healthy lifestyle, which are conducive to cancer prevention (a healthy diet, regular physical activity, smoking avoidance). The next step was to develop a research tool and subjecting it to a pilot study, then evaluating the survey tool based on the data obtained from the pilot study. A professional survey company, specialized in quantitative surveys, with a public opinion research panel was responsible for conducting the survey. The survey was conducted in November 2022 using the Computer Assisted Web Interviewing CAWI (CAWI) technique. Invitations to participate in the survey were sent by mail, phone, SMS, pop-up and web-push to 1,247 randomly selected users of the survey panel. Respondents took part in the study on the basis of informed consent. Before taking part in the study, they were informed about the purpose of the study, the anonymisation of the data, the scientific nature of the use of the results, and the ability to withdraw from the study at any time. If the participants agreed to the above conditions, they confirmed their decision to take part in the study by marking the consents box.

### Research Tool

The research tool was a survey questionnaire consisting of four parts. The first part contained socio-demographic data (e.g., age, place of residence, education level, and financial situation). The second part included questions on the declared frequency of the surveyed preventive behaviours (e.g., a healthy diet, regular physical activity, and avoiding smoking). The respondents rated the frequency of these behaviours on a 5-point scale: 1 - almost never or never and 5 - every day/always, with reverse scores for smoking.

The third part aimed to identify the EPPM groups. The previously published question-construction methodology that was specific to the EPPM model [20, 21] was applied, with the modified list of diseases and health behaviours appropriately for the study objectives. Responses were given on a 5-point Likert scale. Two questions were used to measure the perceptions of cancer threat: “How likely is it that you will develop cancer (lung, colorectal, prostate; covered in separate questions) at some point in the future?” (susceptibility) and “How serious/harmful would the (physical or personal) consequences of this disease be?” (severity). The threat index was calculated as the average of the responses to both questions, separately for each disease. To measure perceived effectiveness, the following questions were asked: “How effective do you think the behaviour is in reducing the risk of the listed diseases?” (response efficacy) and “How would you rate your ability to implement this behaviour to reduce the risk of the following diseases?” (self-efficacy). The efficacy index was calculated as the average of the responses to both questions, separately for each preventive behaviour.

The fourth part of the survey assessed the losses that respondents would potentially suffer as a result of each of the preventive behaviours (for example, the need to find additional time, lack of acceptance from loved ones). It contained the list of 12 losses, four for each healthy behaviour. Responses were assessed on a 5-point Likert scale. A total losses score was calculated as an average of the responses for a given behaviour. All total losses scores had good reliability (above 0.77) as measured by Cronbach’s alpha coefficient.

### Statistical Methods

As recommended in previous studies [15, 16], a threat index and an efficacy index were used to classify respondents into the EPPM groups. By selecting low (below average) and high (above average) levels of perceived efficacy and perceived threat, four EPPM groups were formed. To analyse the differences between groups, a one-way ANOVA for quantitative variables or a chi2 test for categorical variables were used. Microsoft Excel and IBM SPSS were used for the calculations. The significance level was set at p < 0.05. When comparing the EPPM groups, only results statistically significant were included in the tables (therefore Tables 4–6 contain different sets of factors).

## Results

### Characteristics of Respondents

Out of 1,247 invited men, 1,125 met the inclusion criteria for the study. After data verification, 1,000 correctly completed questionnaires were left. The study involved 1,000 men of working age between 18 and 65 years, with a mean age of 41.8 years (Table 1). The majority of participants in the study were employed (81.7%), had a secondary or higher education (82.6%), and assessed their financial situation as average (57.6%). The largest group of respondents (41.7%) lived in rural areas. Almost half (48.7%) of the respondents described their health as good or very good. A total of 560 men declared themselves as smokers, of whom 17.7% smoke daily or 3–4 times a week. The loss indexes for a given healthy behaviours were as follows: for physical activity it was 2.59 (standard deviation 0.94), for healthy eating was 2.79 (standard deviation 0.86), for avoiding smoking was 2.82 (standard deviation 1.00). This means that respondents perceived the losses related to undertaking these behaviours as rather moderate.

**TABLE 1:** Characteristics of respondents (n = 1,000; %) (Warsaw, Poland, 2023).

		Men %
Age	18–29	20.5
	30–39	24.8
	40–49	25.7
	50–59	17.1
	60–65	11.9
Place of residence	Rural areas	41.7
	City >200 K inhabitants	29.0
	City 200–500 K inhabitants	9.1
	City over 500 K inhabitants	20.2
Education	Elementary or junior high school	2.8
	Basic vocational	14.6
	Secondary or post-secondary	42.6
	Higher education	40.0
Employment	Employed (full-time or self-employed)	81.7
	Student	4.0
	Unemployed	4.3
	Pensioner/Retiree	9.4
	Household leader	0.6
Self-assessment of financial situation	Very bad	2.7
	Bad	10.0
	Average	57.6
	Good	25.9
	Very good	3.8
Self-assessment of overall health	Very bad	1.8
	Bad	8.8
	Average	40.7
	Good	41.8
	Very good	6.9
Frequency of healthy behaviours		
Frequency of physical activity [I spend at least 30 min on activities involving moderate to vigorous exercise (e.g., jogging, brisk walking, playing sports, gardening or farm work)]	Almost never or never	11.9
	1–2 times a month	17.7
	3–4 times a week	24.1
	1–2 times a week	31.0
	Everyday	15.3
Frequency of healthy diet [I eat healthily (including eating the recommended amount of vegetables and fruits, limiting the consumption of such products as animal fats, sugar)]	Almost never or never	16.8
	1–2 times a month	16.9
	3–4 times a week	25.0
	1–2 times a week	26.7
	Everyday	14.6
Frequency of smoking	I do not smoke	44.0
	Almost never or never	15.4
	1–2 times a month	10.4
	1–2 times a week	5.6
	3–4 times a week	10.4
	Everyday	17.0

### Perceived Threat of Cancers and Efficacy of Selected Health Behaviours According to the EPPM Dimensions

As presented in Table 2, the level of severity of the consequences resulting from the cancers analysed was rated as meaningful (responses ranging from moderately severe to very severe): 91.4% for prostate cancer, 90.6% for colorectal cancer, and 90.4 for lung cancer. The rate of meaningful susceptibility (responses ranging from moderately likely to very likely) ranged from 44% for lung cancer to 50.3% for colorectal cancer and 51% for prostate cancer.

**TABLE 2:** Perceived threat and efficacy for lung, prostate, and colorectal cancers and selected health behaviours (n = 1,000) (Warsaw, Poland, 2023).

	Perceived susceptibility		Lung cancer (%)	Prostate cancer (%)	Colorectal cancer (%)
Threat	1	Improbable	18.7	16.1	16.5
	2	Unlikely	37.3	32.9	33.2
	3	Moderately likely	31.4	37.8	37.5
	4	Quite likely	10.5	10.3	10.6
	5	Very likely	2.1	2.9	2.2
	Perceived severity				
	1	Harmless	1.1	1.0	1.2
	2	Hardly harmful	8.5	7.6	8.2
	3	Moderately harmful	28.0	31.0	29.4
	4	Quite harmful	43.6	41.9	40.2
	5	Very harmful	18.8	18.5	21.0

	Perceived effectiveness of intervention		Lung cancer	Prostate cancer	Colorectal cancer	
			Avoiding smoking (%)	Healthy diet (%)	Physical activity (%)	Healthy diet (%)
Efficacy	1	Ineffective	4.6	7.2	7.5	5.9
	2	Not very effective	5.5	17.5	15.1	9.1
	3	Moderately effective	19.3	38.9	37.3	32.8
	4	Effective	26.7	24.8	26.3	34.7
	5	Very effective	43.9	11.6	13.8	17.5
	Self-efficacy				
	1	Impossible	7.3	5.1	4.7	4.9
	2	Unlikely	20.9	12.0	18.8	11.1
	3	Moderately likely	27.7	32.5	31.8	29.7
	4	Likely	22.0	34.7	32.5	37.0
	5	Very likely	22.1	15.7	18.2	17.3

For the prevention of lung cancer, smoking avoidance was considered an effective behaviour (responses ranged from moderately effective to very effective) by 89.9% of the respondents. Regular physical activity was considered effective for the prevention of colorectal cancer by 77.4% of the respondents. A healthy diet was considered effective for the prevention of colorectal cancer by 85% of the respondents and for the prevention of prostate cancer by 75.3% of respondents (Table 2). The men surveyed rated their self-efficacy (responses ranged from moderately possible to very possible) in undertaking particular health behaviours as follows: smoking avoidance for lung cancer - 71.8%; regular physical activity for colorectal cancer - 82.5%; a healthy diet for colorectal cancer - 84.0%, and a healthy diet for prostate cancer - 82.9% (Table 2).

The respondents were classified into different groups according to the EPPM: Indifferent, Proactive, Avoidant, and Responsive. In majority of health behaviours the Indifferent groups were the most numerous, followed by Responsive. The exception was a healthy diet for prostate cancer, where the most numerous groups were Indifferent and Proactive (Table 3).

**TABLE 3:** Size of extended parallel process model (EPPM) groups in relation to individual behaviours and malignancies (n = 1,000) (Warsaw, Poland, 2023).

Behaviour	Cancer	Indifferent (%)	Proactive (%)	Avoidant (%)	Responsive (%)
Avoiding smoking	lung cancer	40.5	17.7	15.2	26.6
Physical activity	colorectal cancer	33.4	26.2	13.9	26.5
Healthy diet	colorectal cancer	38.2	21.4	17.4	23.0
Healthy diet	prostate cancer	34.8	26.9	15.4	22.9

### Factors Differentiating the EPPM Groups in Relation to Specific Behaviours and Cancers

The analysis showed that for smoking avoidance in relation to lung cancer, the Responsive group rated their current health status significantly better than the Indifferent group, and the Avoidant group rated the potential losses associated with smoking avoidance as significantly greater than the Proactive group (Table 4). Smoking frequency was found to be non-significant for group differentiation.

**TABLE 4:** Differences between the groups according to the extended parallel process model in relation to reducing tobacco as a lung cancer preventive behaviour (n = 1,000) (Warsaw, Poland, 2023).

Factor	Indifferent (n = 227) M (SD)	Proactive (n = 99) M (SD)	Avoidant (n = 85) M (SD)	Responsive (n = 149) M (SD)	F (p)	Post hoc group comparisons
Health status	3.28 (0.86)	3.38 (0.80)	3.42 (0.75)	3.52 (0.83)	2.72 (0.04)	R>I
Frequency of smoking	3.15 (1.48)	2.82 (1.69)	3.00 (1.72)	2.82 (1.63)	1.57 (0.20)	-
Losses	2.77 (0.94)	2.60 (1.17)	3.01 (0.79)	2.92 (1.05)	3.20 (0.02)	A>P

Note: R, Responsive; A, Avoidant; P, Proactive; I, Indifferent.

The analysis of the groups identified for a healthy diet as a preventive behaviour for prostate cancer showed that the Proactive group had a higher frequency of following a healthy diet, levels of education, current health status, and financial situation than the Indifferent group and a higher frequency of a healthy diet and lower perceived losses than the Avoidant group (Table 5). The men in the Responsive group had a better financial situation and better current health status than men in the Indifferent group, and a higher frequency of a healthy diet than men in the Indifferent and Avoidant groups.

**TABLE 5:** Differences between the groups according to the extended parallel process model in relation to a healthy diet as a prostate cancer preventive behaviour (n = 1,000) (Warsaw, Poland, 2023).

Factor	Indifferent (n = 348) M (SD)	Proactive (n = 269) M (SD)	Avoidant (n = 154) M (SD)	Responsive (n = 229) M (SD)	F (p)	Post hoc group comparisons
Education	3.07 (0.81)	3.30 (0.74)	3.30 (0.77)	3.21 (0.78)	5.48 (<0.001)	P>I; A>I
Financial situation	3.07 (0.79)	3.25 (0.74)	3.16 (0.74)	3.28 (0.77)	4.3 (0.005)	P>I; R>I
Health status	3.29 (0.83)	3.48 (0.81)	3.43 (0.80)	3.60 (0.79)	7.23 (<0.001)	P>I: R>I
Frequency of healthy diet	2.57 (1.25)	3.55 (1.17)	2.64 (1.19)	3.41 (1.23)	45.38 (<0.001)	P>I; P>A; R>I; R>A
Losses	2.78 (0.80)	2.71 (0.95)	2.96 (0.69)	2.80 (0.94)	2.78 (0.04)#	A>P

Note: R, Responsive; A, Avoidant; P, Proactive; I, Indifferent.

The analysis of the groups identified for a healthy diet as a preventive behaviour for colorectal cancer showed that the men in the Proactive and Responsive groups were more likely to use a healthy diet than those in the Indifferent and Avoidant groups (Table 6). The men in the Responsive group were better educated and rated their financial situation as better than those in the Indifferent group. They also rated their health status as better than both the Indifferent and Avoidant groups. In addition, the men in the Avoidant group were older than those in the Indifferent group and rated the losses as higher compared to men in the Proactive and Responsive groups. The men in the Proactive group rated their financial situation and current health status as better compared to men in the Indifferent group.

**TABLE 6:** Differences between the groups according to the extended parallel process model in relation to a healthy diet and physical activity as a colon cancer preventive behaviour (n = 1,000) (Warsaw, Poland, 2023).

Factor	Indifferent (n = 382) M (SD)	Proactive (n = 214) M (SD)	Avoidant (n = 174) M (SD)	Responsive (n = 230) M (SD)	F (p)	Post hoc group comparosons
Healthy diet						
Age	40.92 (12.28)	41.07 (13.29)	43.94 (12.35)	42.27 (12.66)	2.64 (0.05)	A>I
Education	3.09 (0.79)	3.23 (0.76)	3.22 (0.84)	3.33 (0.75)	5.13 (0.002)	R>I
Financial situation	3.04 (0.79)	3.27 (0.68)	3.18 (0.70)	3.34 (0.80)	9.35 (<0.001)	P>I; R>I
Health status	3.28 (0.80)	3.52 (0.84)	3.40 (0.77)	3.62 (0.81)	9.39 (<0.001)	P>I: R>I: R>A
Frequency of healthy diet	2.59 (1.22)	3.55 (1.25)	2.65 (1.22)	3.60 (1.12)	53.24 (<0.001)	P>I; P>A; R>I; R>A
Losses	2.82 (0.82)	2.71 (0.99)	3.01 (0.65)	2.66 (0.91)	6.46 (<0.001)	A>P; A>R
Physical activity						
Factor	Indifferent (n = 334)	Proactive (n = 262)	Avoidant (n = 139)	Responsive (n = 265)	F(p)	Post hoc
Place of residence	2.07 (1.14)	2.03 (1.14)	2.35 (1.24)	2.00 (1.08)	3.12 (0.03)	A>R
Education	3.11 (0.80)	3.18 (0.76)	3.18 (0.83)	3.34 (0.76)	4.37 (0.005)	R>I
Financial situation	3.06 (0.76)	3.19 (0.76)	3.16 (0.74)	3.33 (0.77)	6.07 (<0.001)	R>I
Health status	3.30 (0.80)	3.45 (0.86)	3.33 (0.80)	3.63 (0.79)	8.77 (<0.001)	R>I; R>A
Physical activity	2.84 (1.21)	3.43 (1.12)	2.61 (1.24)	3.47 (1.14)	28.94 (<0.001)	P>I; R>I; P>A; R>A

Note: R, Responsive; A, Avoidant; P, Proactive; I, Indifferent.

The analysis of the EPPM groups regarding physical activity as a preventive behaviour for colorectal cancer showed that men in the Responsive and Proactive groups were more physically active than those in the Indifferent and Avoidant groups (Table 6). Those belonging to the Responsive group had a better financial situation and education than those from the Indifferent group. The men in the Avoidant group lived in larger towns and cities than those in the other groups.

## Discussion

The main objective of the study was to examine how Polish men perceive the threat (severity and susceptibility) of three types of cancer (colon, lung and prostate) and the efficacy (response efficacy and self-efficacy) of three preventive behaviours (healthy diet, physical activity, smoking cessation).

We found that Polish men believe that lung, prostate and colon cancer would be harmful to them. About half of the respondents considered the occurrence of prostate and colon cancer as probable, but estimated that lung cancer is the least likely to develop. This is similar to Chen’s study, in which respondents rated the risk of lung cancer as quite low [22]. The question is that how much of this result is due to inadequate knowledge and how much is due to specific, persistent mechanisms in smokers to process information about the risk, i.e., by ignoring, downplaying, or underestimating it. It cannot be ruled out also that asking a question directly about one’s own risk may have triggered a situational defence mechanism in the form of optimistic bias and a consequent underestimation of that risk. It seems likely that the significant difference in lung cancer risk perception revealed in the results may be due to a lack of knowledge about the harms of smoking. As studies show, smokers’ knowledge of the risks of smoking is still insufficient [23, 24]. One of the recent Polish studies showed that smokers are less likely to be aware of the health consequences of smoking compared to non-smokers [25]. Moreover, a 2018 study in Poland highlights the need to improve knowledge of the risks of active and passive smoking among socially disadvantaged populations [26]. In addition, Dutch researchers have drawn attention to the necessity to improve knowledge of the harms of smoking among smokers with low education [27]. Respondents perceived smoking avoidance to be the most effective intervention for lung cancer prevention, while considering this to be the least feasible strategy to implement. The disparity between severity and susceptibility was noted to be greatest in the lung cancer risk assessment compared to the other cancers.

The analysis of the EPPM group sizes indicated a significant numerical advantage for the Indifferent group, characterised by a low sense of lung cancer threat as well as a low efficacy of smoking avoidance. What is most surprising, however, is that belonging to the EPPM groups did not differentiate smoking frequency among the respondents declaring to smoke at all, representing 56% of the respondents. According to current population data, about 30% of Polish men smoke cigarettes (24% are compulsive smokers and about 6% are occasional smokers) [28]. Out of these, 42% have not tried to quit smoking and 58% have tried to quit, but failed [29]. These results are consistent with a study by Ziebarth, showing that smokers who self-report that they do not plan to quit smoking are significantly more likely to underestimate their lifetime lung cancer risk [30, 31]. The identified groups differed in their assessment of current health status and losses associated with reducing smoking. The Responsive group rated their health status as better than the Indifferent group, and the Avoidant group rated the potential losses from reducing smoking as greater than the Proactive group. Thus, efficacy seems to be a key component for the last result and is indicated by other authors as an important factor in the willingness to change behaviour [32]. Referring to our results, perceived self-efficacy turns out to be essential.

In the study group, prostate cancer was considered the most likely and most severe in terms of consequences; however, in reference to a healthy diet, the most numerous groups according to the EPPM classification were Indifferent and Proactive groups, both with a low perceived risk of this cancer. This contrasts with the fact that men in Western countries have a much higher incidence of prostate cancer than men from Asian countries. One possible reason is a difference in their lifestyles, especially in diet. It was confirmed that men who are already at a higher risk due to age, race, or genetics can reduce their risk of prostate cancer by following a healthy diet [33, 34]. Research among men indicates that the knowledge of prostate cancer is satisfactory. They consider it to be a common disease among men and to have serious consequences, but perceive the risk of the disease to be low, which the authors of previous studies linking this to low screening attendance [35, 36]. Analysing the differences between the Proactive and Indifferent groups, it can be seen that men in the Proactive group were more likely to have a healthy diet, but also had a higher level of education, better health, and a better financial situation. The other studies indicate that men who declare satisfactory health status, live in an urban area, and have a higher education level are more likely to participate in preventive activities [37]; however, taking into account recent research and the results we obtained for Polish men, it appears that the efficacy component is a factor that may influence behaviour change and the adoption of a healthy diet. Therefore, the most valuable programmes seem to be those that provide men with information not only increasing their knowledge about prostate cancer, but also their self-efficacy and, consequently, adherence to healthy behaviour [38].

The intervention considered effective and the most likely to participate by respondents was a healthy diet for colorectal cancer; however, when analysing the size of each group, the largest number of men were in the Indifferent and Responsive groups. The men in the Responsive group rated their education, financial situation, and health status as better compared to men in the Indifferent group for both a healthy diet and physical activity in colorectal cancer. According to the previous studies, the level of education can influence both self-efficacy and perceptions of intervention efficacy [39]. In addition, the men in the high-efficacy groups were more likely to engage in preventive behaviour compared to men in the low-efficacy groups. Other studies showed that greater self-efficacy in participating in a healthy diet correlates positively with checking product labels, limiting fast food, and eating more dark green vegetables [40]. One recent study indicates that people with low self-efficacy are more likely to experience more eating behaviour problems compared to those with high self-efficacy [41]. Additionally, the men from the group with a high threat but low efficacy (avoidant) rated the potential losses from implementing a healthy diet as higher compared to men from the groups with a high efficacy (Responsive and Proactive). The men in the Avoidant group were significantly older compared to men in the Indifferent group, suggesting that when the risk of disease increases with age and the range of ways of coping with problems decrease, the avoidance of confronting health problems may become the most available coping strategy.

In summary, across all of the behaviours studied, the groups characterised by high efficacy (Responsive and Proactive) showed a higher frequency of preventive behaviours in relation to the cancers studied. The exception was lung cancer. This result is puzzling in the context of the accuracy of predictions of a given behaviour based on the EPPM groups. It may suggest that, in the case of smoking avoidance, factors other than perceptions of cancer threat and behaviour efficacy are central. They would need to be explored in future studies to explain this phenomenon (e.g., level of distress, a person’s psychological wellbeing, and individual psychosocial resources) [42]; however, these results suggest the need for interventions aimed at increasing the perceived risk of smoking in the context of lung cancer and those aimed at increasing men’s self-efficacy in reducing smoking. According to health psychologists, people are more likely to engage in healthy behaviours if they have confidence in their ability to carry out these behaviours successfully [42].

### Limitations of the Study

The data analysed are declarative, especially in terms of self-efficacy and the frequency of behaviours.

More objective measures of actual behaviour using newer technologies are needed such as mobile applications or wearable devices, which might increase measurement accuracy [43]. Despite the study being conducted on a representative group, it was noted that the declared population of smokers in the study was much larger than other data sources for Poland suggest. It cannot be excluded that the over-representation of this group is due to the criteria adopted for classifying smokers, e.g., compulsive smokers versus any frequency of smoking. All but those who answered “I don’t smoke” were included in our study. When practising other behaviours, such radical criteria were not applied. Due to this selection bias, the results should be interpreted with caution. Another problem is the criterion for selecting EPPM groups. We used the mean as a cut-off point for selecting low/high threat and low/high efficacy respondents, which has some advantages and disadvantages. Using this criterion, as in previous studies, allowed us to maintain consistency with them. Due to the different distributions of the threat and efficacy variables this method did not produce equal groups (as seen in our results) and may at least partially reflect the actual group sizes. Other criteria could be considered, such as the median, which increases the chance of obtaining similarly sized groups, or the interquartile deviation, which may sharpen the differences between groups but results in smaller group sizes. It would be worthwhile to conduct separate studies comparing these methods. In addition, the study focused on selected preventive behaviours for the cancers studied, which do not include all possible modifiable behaviours aimed at reducing cancer risk. The EPPM-based analysis included a limited number of factors that would allow for a more extensive characterisation of the groups. Family support, which is considered one of the predictors of preventive behaviour, was not included in the study. Future research should include a broader range of factors determining EPPM segmentation, e.g., multimorbidity, which may impact both on perceived cancer threat and efficacy of health behaviours. From the public health perspective, the priority seems to recognize factors increasing the chance of belonging to the Responsive and Proactive EPPM groups, with the higher frequency of health behaviours.

### Conclusions

In particular, the need to implement interventions aimed at: (1) increase the perceived risk of smoking in the context of lung cancer incidence, (2) increase men’s self-efficacy in smoking cessation and (3) reduce the level of perceived losses from undertaking a healthy diet and smoking cessation (especially among men with low efficacy). It should be taken into account that in the case of interventions aimed at increasing efficiency, they will be directed to people with a worse financial situation and lower education.
