# Supplementary material for: Smoking Avoidance, Physical Activity and Diet as Preventative Behaviours for Lung, Prostate and Colorectal Cancer - A Comparison of the Extended Parallel Process Model Groups
Source: Int J Public Health. 2025 Feb 4;70:1607278. doi: 10.3389/ijph.2025.1607278 (PMC11832310; doi:10.3389/ijph.2025.1607278)
Supplement: Supplementary file 1 [file DataSheet1.docx]

Supplementary

Figure S1 Structure of Extended Parallel Process Model based on Witte 1982 [15] (Poland,2024)

|  | High efficacy | Low efficacy |
| --- | --- | --- |
| High threat | Responsive | Avoidant |
| Low threat | Proactive | Indifferent |

S2 Questionnaire

I

1. What year were you born? ……………………………….
2. What is your place of residence::
3. Countryside
4. City up to thousand inhabitants
5. City 200 to 500 thousand inhabitants
6. City over 500 thousand inhabitants
7. What kind of education do you have:
8. Elementary or junior high school
9. Basic vocational
10. Secondary or post-secondary education
11. Higher education (bachelor's degree and above)
12. What is your status in the labor market:
13. Working (employed full-time or self-employed)
14. Student
15. Unemployed
16. Pensioner
17. What type of work do you do:
18. Physical
19. Mental
20. Mixed
21. How do you assess your financial situation:
22. Bad
23. Average
24. Good
25. Very good
26. How would you rate your health condition::
27. Very bad
28. Bad
29. Average
30. Good
31. Very good

Frequency of use of preventive behavior

|  |  | 1 almost never or never | 2  seldom | 3  From time to time | 4  often | 5 almost always or always |
| --- | --- | --- | --- | --- | --- | --- |
| Healthy diet | I eat a lot of vegetables in and fruits |  |  |  |  |  |
|  | I limit the consumption of such products as animal fats, sugar |  |  |  |  |  |
|  | I take care of proper nutrition |  |  |  |  |  |
|  | I eat whole-grain bread |  |  |  |  |  |
| Physical activity | I dedicate at least 30 minutes every day to activities involving moderate to vigorous exercise (e.g. jogging, brisk walking, playing sports, gardening or farm work) |  |  |  |  |  |
|  |  | 5 almost never or never | 4  1-2 times a month | 3  1-2 times a week | 2  3-4 times a week | 1  daily |
| Limiting smoking | I am currently smoking |  |  |  |  |  |

Risk assessment

1. How likely is it that you will develop the following conditions at some point in the future? 1 – unlikely; 2 – very unlikely; 3 – moderately likely; 4 – likely; 5 – very likely (perceived susceptibility)

|  | 1 unlikely | 2 | 3 | 4 | 5 very likely |
| --- | --- | --- | --- | --- | --- |
| Lung cancer |  |  |  |  |  |
| Prostate cancer |  |  |  |  |  |
| Colorectal cancer |  |  |  |  |  |

1. How serious/harmful would the consequences be for you (physical, e.g. pain/treatment/hospitalization and personal: self-esteem, relationships with loved ones) of getting the following diseases (1- harmless; 2- slightly harmful; 3- moderately harmful; 4- harmful; 5- very harmful) (perceived severity)

|  | 1 harmless | 2 | 3 | 4 | 5 very harmful |
| --- | --- | --- | --- | --- | --- |
| Lung cancer |  |  |  |  |  |
| Prostate cancer |  |  |  |  |  |
| Colorectal cancer |  |  |  |  |  |

Efficacy Assessment

Intervention efficacy

1. In your opinion, to what extent is a healthy diet (rich in vegetables, fruits, whole grain products, low in animal fats) effective in reducing the risk of colon and prostate cancer? (1-ineffective; 2 - slightly effective; 3- moderately effective; 4 - effective; 5-very effective)

|  | 1 Ineffective | 2 | 3 | 4 | 5- very effective |
| --- | --- | --- | --- | --- | --- |
| Colorectal cancer |  |  |  |  |  |
| Prostate cancer |  |  |  |  |  |

1. In your opinion, to what extent is regular physical activity effective in reducing the risk of colon cancer? (1-ineffective; 2 - slightly effective; 3 - moderately effective; 4 - effective; 5 - very effective)

|  | 1- ineffective | 2 | 3 | 4 | 5 - very effective |
| --- | --- | --- | --- | --- | --- |
| Colorectal cancer |  |  |  |  |  |

1. In your opinion, to what extent is avoiding smoking effective in reducing the risk of lung cancer? (1-ineffective; 2-slightly effective; 3-moderately effective; 4-effective; 5-very effective)

|  | 1- Ineffective | 2 | 3 | 4 | 5- very effective |
| --- | --- | --- | --- | --- | --- |
| Lung cancer |  |  |  |  |  |

Self Effectivneess

1. How do you assess your ability to adopt a healthy diet (rich in vegetables, fruits, whole grain products, low in animal fats) in order to reduce the risk of colon cancer and prostate cancer? (1-impossible; 2- unlikely; 3- moderately possible; 4- possible; 5- very possible)

|  | 1- impossible | 2 | 3 | 4 | 5- very possible |
| --- | --- | --- | --- | --- | --- |
| Colorectal cancer |  |  |  |  |  |
| Prostate cancer |  |  |  |  |  |

1. How do you assess your ability to implement regular physical activity in your life in order to reduce the risk of developing colon cancer? (1 - impossible; 2 - unlikely; 3 - moderately possible; 4 - possible; 5 - very possible)

|  | 1- impossible | 2 | 3 | 4 | 5- very possible |
| --- | --- | --- | --- | --- | --- |
| Colorectal cancer |  |  |  |  |  |

1. How do you assess your ability to implement avoiding smoking in order to reduce the risk of lung cancer? (1-impossible; 2- unlikely; 3- moderately possible; 4- possible; 5- very possible)

|  | 1- impossible | 2 | 3 | 4 | 5- very possible |
| --- | --- | --- | --- | --- | --- |
| Lung cancer |  |  |  |  |  |

Generalized Self-Efficacy Scale (GSES)

Below are several statements relating to various personal characteristics. After reading them, please relate the statement to yourself and determine if they are true or not by writing your answer on a four-point scale:

| LP |  | No | Rather no | Rather yes | Yes |
| --- | --- | --- | --- | --- | --- |
| 1 | I am always able to solve difficult problems if I try hard enough |  |  |  |  |
| 2 | If someone opposes me, I have ways to achieve what I want |  |  |  |  |
| 3 | I find it easy to stick to my goals |  |  |  |  |
| 4 | I am confident that she/he could effectively deal with unexpected events |  |  |  |  |
| 5 | Thanks to their ingenuity to cope with the unexpected situations |  |  |  |  |
| 6 | I can solve most problems if I put in enough enough effort |  |  |  |  |
| 7 | I can remain calm in the face of difficulties because I can rely on my coping skills |  |  |  |  |
| 8 | When I struggle with a problem, I usually find a few solutions |  |  |  |  |
| 9 | When I am in a troublesome situation, I generally know what to do |  |  |  |  |
| 10 | Regardless of what I face, I can handle it |  |  |  |  |

Losses related to the implementation of health behaviors

|  | 1  completely irrelevant | 2  Rather unimportant | 3  Moderately  significant | 4  Rather  significant | 5  Highly  significant |
| --- | --- | --- | --- | --- | --- |
| If I introduced the principles of healthy eating: | | | | | |
| I would waste a lot of time preparing healthy meals |  |  |  |  |  |
| I would have to give up many tasty foods that I enjoy eating/drinking |  |  |  |  |  |
| My friends would laugh at me |  |  |  |  |  |
| I would go hungry |  |  |  |  |  |
| Other: |  |  |  |  |  |
| If I introduced regular physical activity: | | | | | |
| I would have to force myself to do physical exercise that I don't like |  |  |  |  |  |
| I would have to find extra time that I don't have, at the expense of other activities in my free time |  |  |  |  |  |
| my muscles would hurt after exercising |  |  |  |  |  |
| my loved ones would laugh at me |  |  |  |  |  |
| Other: |  |  |  |  |  |
| If I reduce smoking: | | | | | |
| I might gain weight |  |  |  |  |  |
| I would have to give up something I like |  |  |  |  |  |
| I would feel nervous/undermined |  |  |  |  |  |
| My social life would suffer |  |  |  |  |  |
| Other: |  |  |  |  |  |
